# The Role of Klotho in Oral and Maxillofacial Diseases: Mechanisms and Research Progress

**DOI:** 10.3390/biom15050624

**Published:** 2025-04-27

**Authors:** Shiqi Lin, Bozhao Wang, Jian Li

**Affiliations:** 1 School of Medicine, Xiamen University, Xiamen 361102, China; kikiii@stu.xmu.edu.cn (S.L.); bobby@stu.xmu.edu.cn (B.W.); 2 Department of Stomatology, Xiang’an Hospital of Xiamen University, Xiamen 361102, China

**Keywords:** Klotho, oral and maxillofacial diseases, oxidative stress, inflammation, apoptosis, anti-aging

## Abstract

Klotho, an anti-aging protein, has been extensively studied in systemic conditions such as chronic kidney disease and cardiovascular disorders. In recent years, its pivotal protective role and clinical significance in various oral and maxillofacial diseases have been increasingly demonstrated. It has been demonstrated that Klotho regulates oxidative stress, apoptosis, inflammation, and fibrosis via multiple molecular signaling pathways, including Nrf2, NF-κB, PI3K/Akt/FoxO1, insulin/IGF-1, FGF/FGFR, and Wnt/β-catenin. Consequently, these regulatory effects have been observed in conditions such as periodontitis, oral squamous cell carcinoma, malignant salivary gland tumors, oral submucous fibrosis, etc. Moreover, the decreased expression or dysfunctional activity of Klotho is frequently associated with the onset and progression of these diseases. This study provides a comprehensive review of the underlying mechanisms and recent advances in Klotho research within the realm of oral and maxillofacial diseases, offering novel perspectives for future basic and clinical investigations.

## 1. Introduction

Klotho is an anti-aging protein that has been the subject of considerable attention due to its potential to regulate the aging process, maintain metabolic homeostasis, and exert anti-inflammatory effects [1]. The protein is expressed in three distinct forms: the full-length transmembrane, the soluble, and the secreted variants, which are all implicated in regulating key physiological processes, including calcium–phosphorus metabolism, antioxidant responses, and apoptosis [2]. In recent years, the association between Klotho and various systemic diseases has been increasingly explored, particularly in relation to its role in oral and maxillofacial disorders.

Oral and maxillofacial diseases, such as periodontitis, oral squamous cell carcinoma, malignant salivary gland tumors, and oral submucous fibrosis, are common conditions that affect global human health. These disorders are frequently characterized by pathological processes, including oxidative stress, inflammation, apoptosis, and fibrosis, wherein Klotho has been demonstrated to play multifaceted regulatory roles [3,4,5]. The present study is designed to systematically investigate the molecular mechanisms by which Klotho influences oral and maxillofacial diseases, with the aim of providing novel therapeutic insights.

## 2. Overview and Basic Functions of Klotho

Klotho was initially discovered in 1997 through investigations conducted on *transgenic mouse* models by Kuro-o and colleagues [6]. The gene was named after Clotho—one of the three Fates in Greek mythology responsible for spinning the thread of life. Klotho is expressed in the kidneys, parathyroid gland, and heart. In the craniofacial region, Klotho expression has been detected in bone marrow and periodontal ligament stem cells (PDLSCs) [7]. The protein has attracted significant attention because of its crucial roles in modulating aging processes, maintaining metabolic balance, and mediating anti-inflammatory effects [6,8,9].

The *Klotho* gene comprises five exons and encodes a type I single-pass transmembrane protein of 1014 amino acids. The intracellular domain is notably short and lacks any known functional motifs, whereas the extracellular domain is composed of two tandem repeat sequences, designated as KL1 and KL2, which exhibit limited homology.

The protein encoded by the Klotho gene exists in three major forms: full-length transmembrane Klotho, soluble Klotho, and secreted Klotho. Full-length transmembrane Klotho is predominantly expressed in the kidney and parathyroid gland, where its KL1 and KL2 domains serve as co-receptors for various ligands. Soluble Klotho is generated by the cleavage of the full-length protein at a β-site, thereby releasing the KL1 domain, which is then capable of exerting systemic effects through the circulatory system. Similarly, secreted Klotho, which also contains the KL1 domain, is functionally analogous to soluble Klotho [2]. By studying the transfection of bone marrow mesenchymal stem cells (BMSCs) with *Klotho* gene recombinant adenovirus (BMSCs-Klotho) and *C57 BL/6 mouse* models, it was found that circulating soluble Klotho is recognized as an endocrine factor that mediates diverse functions, including the regulation of ion channels, antagonism of insulin signaling, inhibition of Wnt signal transduction, suppression of cellular senescence, and provision of antioxidative effects [10].

## 3. The Core Functional Network of Klotho

Klotho, as a multifunctional regulatory protein, is recognized for its critical role in systemic diseases through the integration of signaling networks related to aging, oxidative stress, metabolism, and inflammation. Its core functional network comprises multidimensional regulatory mechanisms, including those governing aging, antioxidant defense, calcium–phosphorus metabolism, inflammatory responses, and key signaling pathways. Telomerase activity is modulated to delay stem cell exhaustion, nuclear factor erythroid 2–related factor 2 (Nrf2) is activated to enhance antioxidant capacity, and metabolic homeostasis is maintained in cooperation with the fibroblast growth factor (FGF) family. Concurrently, pro-aging and pro-inflammatory signaling cascades are suppressed, thereby establishing the molecular foundation for Klotho’s anti-aging effects, its mitigation of metabolic diseases, and its prevention of tissue fibrosis. This multifaceted network underscores the potential of Klotho as a target for disease intervention.

### 3.1. Aging Regulatory Mechanisms

#### 3.1.1. Regulation of Telomerase Activity

It has been demonstrated that telomere dysfunction is closely associated with aging-related diseases. For instance, telomere shortening has been reported as a common biomarker for aging, immunosenescence, and autoimmune disorders [11]. By studying *Klotho null (KspKL−/−) mice*, it has further been observed that Klotho deficiency leads to the transcriptional repression of key telomerase components such as telomeric repeat binding factor 1 (TERF1) and telomerase reverse transcriptase (TERT), which subsequently results in reduced telomerase activity and stem cell exhaustion. This process is mediated by the interactive regulation of the transforming growth factor-β(TGFβ), insulin, and Wnt signaling pathways [12]. Notably, the decline in telomerase activity can be effectively counteracted by cycloastragenol (CAG) in *female Institute of Cancer Research (ICR) mice*, suggesting that the anti-aging effects of Klotho are mediated via the modulation of telomere length and telomerase activity. In addition, it was demonstrated that CAG successfully restored beta-Klotho (β-Klotho, KLb) expression levels in an ovarian aging model, thereby confirming the pivotal role of the Klotho–telomerase regulatory axis in reproductive system aging [1]. Collectively, these findings indicate that Klotho is involved in the regulation of cellular aging through the modulation of telomerase activity.

#### 3.1.2. DNA Damage Repair Pathways

Recent studies have demonstrated that the Klotho protein plays a critical regulatory role in maintaining genomic stability and delaying cellular senescence. It has been reported that, when transformed *human microglial cells* (HMC3; ATCC CRL-3304) were exposed to cigarette smoke or aerosol, DNA damage was induced and the expression of β-Klotho was reduced [13]. β-Klotho is an aging biomarker associated with aging and cyclicity [1]. Nakayama et al. used *human renal proximal tubular epithelium (HK-2) cells* and *α-Klotho KO/Jcl transgenic mice* models to find that the knockdown of Klotho significantly enhances DNA damage induced by ionizing radiation (IR) [14]. Additionally, in *siRNA-treated ICR male mice* and *Nrf 2-knockout ICR male mice*, it has been revealed that, during aging, the hypermethylation of the Nrf2/Klotho promoter mediated by DNA methyltransferase (DNMT)1, DNMT3a, DNMT13b leads to the suppression of its expression, an effect that can be reversed by the DNMT inhibitor SGI-KLOT 1072 via demethylation [15]. These studies provide compelling evidence that Klotho plays a central regulatory role in both DNA damage repair and anti-aging processes.

### 3.2. Regulation of Oxidative Stress

#### 3.2.1. Nrf2/ARE Signaling Axis

Nrf2 is recognized as a key transcription factor responsible for regulating the cellular antioxidant response. Under physiological conditions, Nrf2 is ubiquitinated and degraded as a result of binding to Kelch-like ECH-associated protein 1 (Keap1). By exerting antioxidant effects, Klotho is found to reduce the production of reactive oxygen species (ROS) under oxidative stress, thereby promoting the dissociation of the Keap1–Nrf2 complex. Consequently, Nrf2 is released, translocated into the nucleus, and bound to the antioxidant response element (ARE) to induce the expression of antioxidant genes such as superoxide dismutase (SOD), NAD(P)H quinone dehydrogenase 1 (NQO1) and heme oxygenase-1 (HO-1). Thus, it is suggested that the antioxidant response is regulated by Klotho through the activation of the Nrf2 signaling pathway and the subsequent upregulation of antioxidant enzyme expression [8,16,17].

#### 3.2.2. Mitochondrial Function Protection

Mitochondrial function has been shown to be regulated by Klotho via the negative modulation of the PI3K/Akt/FoxO1 pathway, thereby enhancing the antioxidant capacity of *human periodontal ligament stem cells* (hPDLSCs) and protecting them from H_2_O_2_-induced oxidative stress [16]. As mitochondria serve as dual regulatory centers for ROS generation and clearance, they are highly susceptible to oxidative damage, which may result in imbalances in membrane potential and trigger mitochondria-dependent apoptotic pathways.

### 3.3. Regulation of Calcium and Phosphate Metabolism

Fibroblast growth factor-23 (FGF-23) is predominantly derived from bone and expressed in osteoblasts and osteocytes. As an essential co-receptor, the single transmembrane protein alpha-Klotho (α-Klotho) forms complexes with the fibroblast growth factor receptor (FGFR)1c/3c/4 through its extracellular KL1/KL2 domain. Within this complex, the N-terminal domain of FGF23 is bound specifically to the extracellular region of the FGFR, while the C-terminal domain is engaged with the KL1/KL2 domain of α-Klotho to form the FGF23–FGFR–α-Klotho terpolymer, which serves as the critical molecular foundation for initiating downstream signal transduction [18]. In contrast, FGF21 has been found to bind FGFR1 via β-Klotho, resulting in the activation of the SIRT1/mTOR pathway, which promotes fatty acid oxidation, antioxidant defense, and metabolic regulation [19]. Through collaborative interactions with endocrine FGF molecules such as FGF-23 and FGF21, Klotho is positioned as a co-receptor within the FGFR signaling system and plays a central role in regulating phosphate homeostasis, lipid metabolism, antioxidant defenses, and anti-inflammatory responses.

### 3.4. Inflammatory Regulation Network

Nuclear factor-κB (NF-κB) is a pivotal transcription factor in the inflammatory response. Klotho has been shown to prevent the activation of NF-κB by reducing oxidative stress and the production of inflammatory mediators such as tumor necrosis factor-α (TNF-α), interleukin-6 (IL-6), and interleukin-1β (IL-1β). In addition, the phosphorylation and degradation of the NF-κB inhibitor (IκB) are suppressed by Klotho through both direct and indirect mechanisms, thereby reducing the nuclear translocation of NF-κB. Consequently, inflammation is mitigated via the regulation of the NF-κB signaling pathway and the inhibition of proinflammatory factor expression [8,20].

Moreover, Nrf2 is regarded as an anti-inflammatory agent through its inhibition of NF-κB activity. It has been reported that cigarette smoke impairs Nrf2 translocation and activates the NF-κB pathway in HMC 3 cells (ATCC CRL-3304), with increased NF-κB expression further suppressing Nrf2 [13]. Thus, an antagonistic relationship between Nrf2 and NF-κB is evident under conditions of oxidative stress and inflammation. By activating Nrf2, Klotho is able to reduce oxidative stress, thereby indirectly inhibiting the activation of NF-κB. Simultaneously, the direct inhibition of NF-κB by Klotho diminishes inflammation and further alleviates oxidative stress, thereby supporting Nrf2 activity [8,9].

### 3.5. Regulation of the Wnt/β-Catenin Signaling Pathway

Klotho functions as an endogenous antagonist of the Wnt/β-catenin signaling pathway by specifically binding to the Wnt1 ligand and competitively inhibiting its interaction with the low-density lipoprotein receptor-related protein 6 (LRP6) co-receptor on the cell surface, thus reducing β-catenin activation. Based on a *C57BL/6 mice* model of renal injury and recombinant *human* Klotho protein intervention in *human proximal renal tubular epithelial cells (HKC-8)*, it has been demonstrated that Klotho attenuates Wnt1-induced mitochondrial damage and ROS generation, which in turn improves mitochondrial function and inhibits fibrosis and senescence in tubular cells. Consequently, by inhibiting the Wnt1/β-catenin signaling pathway, Klotho significantly reduces fibrosis, cellular senescence, and mitochondrial injury, thereby protecting renal function. This mechanism holds significant potential for the treatment of chronic kidney disease and other fibrotic disorders [21].

### 3.6. Insulin/IGF-1 Signaling Pathway

The insulin/IGF-1 signaling pathway is a fundamental mechanism that regulates cell growth, metabolism, and aging. It has been reported that the overactivation of this pathway exacerbates oxidative stress and accelerates cellular senescence [19]. Klotho has been shown to downregulate the insulin/IGF-1 signaling pathway, thereby reducing PI3K/AKT pathway activity and enhancing activation of the transcription factor FoxO [22]. This modulation promotes the expression of antioxidant genes, which in turn alleviates oxidative stress. Moreover, Klotho has been demonstrated to delay age-related functional decline in *transgenic mice* by targeting and negatively regulating the insulin/IGF-1 signal transduction network [23]. This dual role in metabolic regulation, antioxidation, and anti-aging renders Klotho an important potential target for the treatment of metabolic diseases and the delay of aging.

## 4. Mechanisms of Klotho in Oral and Maxillofacial Diseases

**Figure 1:**
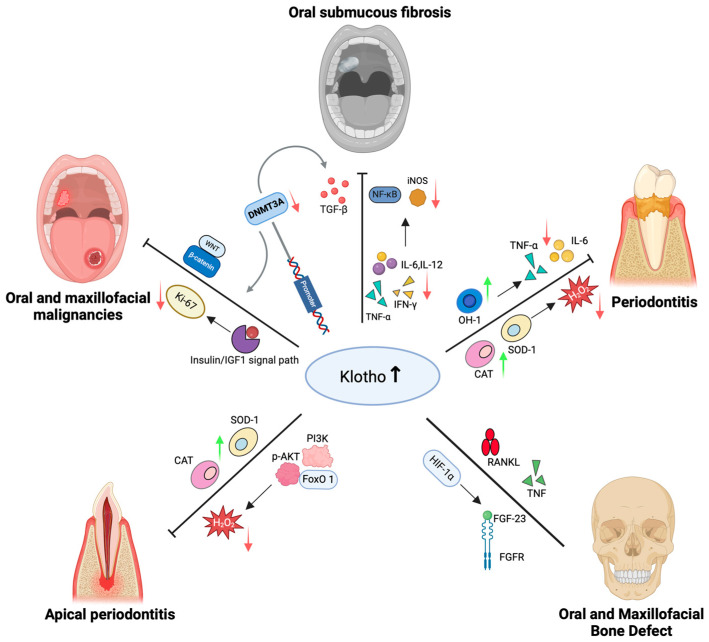
Klotho in oral and maxillofacial diseases. When Klotho levels increase, it exerts anti-inflammatory, antioxidant, and anti-apoptotic effects by activating Nrf2, inhibiting NF-κB, and regulating several pathways such as PI3K/Akt/FoxO1, TGF-β, IGF-1/insulin, and FGF. This process reduces pro-inflammatory factors, including TNF-α, IL-6, and IL-12, while upregulating antioxidant enzymes such as SOD, CAT, HO-1, and NQO1. In periodontal and periapical diseases, Klotho suppresses oxidative stress and apoptosis, maintains stem cell activity, and mitigates inflammation. In oral mucosal fibrosis, Klotho interacts with the IL-12/NF-κB/iNOS and Nrf2 axes to inhibit fibrosis. In oral and maxillofacial tumors, the transcriptional levels of Klotho are influenced by promoter hypermethylation and the Wnt/β-catenin and IGF-1 axes, subsequently regulating tumor proliferation and invasion. In bone metabolism, Klotho interacts with signaling molecules such as FGF-23 and RANKL/TNF, modulating the balance between osteogenesis and osteoclastogenesis, thereby maintaining bone homeostasis. In conclusion, Klotho, as a key anti-aging protein, plays a critical role in the occurrence, progression, and repair of various oral and maxillofacial diseases, with potential diagnostic and therapeutic value.

Although the regulatory mechanisms of Klotho in systemic conditions such as chronic kidney disease and cardiovascular disorders have been well-established, its specific role in oral and maxillofacial diseases has not been systematically elucidated. Recent investigations have implied that Klotho is involved in the pathogenesis of various oral and maxillofacial diseases, including periodontitis, periapical periodontitis, oral submucous fibrosis, and oral and maxillofacial malignancies. This section is dedicated to delineating the molecular regulatory characteristics and underlying mechanisms of Klotho in oral diseases, thereby providing novel perspectives for targeted therapeutic interventions (Figure 1).

### 4.1. Role of Klotho in Periodontitis

Epidemiological surveys have indicated that periodontitis ranks as the sixth-most prevalent disease in humans, affecting more than 60% of Chinese adults to varying degrees [24]. The onset and progression of periodontitis have been attributed to the interaction between bacterial biofilms and the host immune response. Factors such as oxidative stress, systemic infection or inflammation, aberrant tissue metabolism, cellular senescence, and apoptosis have been demonstrated to promote the progression of periodontitis by influencing host immunity or altering the periodontal microbiota [25,26]. PDLSCs, which possess multipotent capabilities, play a critical role in the remodeling of periodontal support tissues and have recently emerged as promising seed cells for periodontal defect repair [27].

In periodontitis, Klotho has been reported to exert anti-inflammatory effects by activating the Nrf2 signaling pathway while concurrently inhibiting the NF-κB pathway. As a result, the release of pro-inflammatory cytokines such as tumor necrosis factor (TNF)-α and IL-6 is reduced, thereby ameliorating periodontal inflammation [28,29]. The activation of Nrf2 has also been demonstrated to upregulate the expression of antioxidant genes, such as *SOD-1*, *CAT*, *NQO1*, and *HO-1*, which further enhances anti-inflammatory responses and confers protection to periodontal tissues [28]. Moreover, it has been demonstrated that Klotho modulates the PI3K/Akt/FoxO1 signaling pathway, leading to an increase in the activity of antioxidant enzymes such as SOD and CAT, thereby mitigating H_2_O_2_-induced oxidative stress and preserving both the viability and differentiation capacity of hPDLSCs [16,29]. In addition, Klotho has been showed to significantly reduce H_2_O_2_-induced apoptosis in hPDLSCs by upregulating anti-apoptotic proteins (e.g., Bcl-2) and downregulating pro-apoptotic proteins (e.g., Bax and Caspase-3) [16,29]. Furthermore, by modulating the expression of uncoupling protein 2 (UCP2), Klotho protects mitochondrial function and reduces ROS generation, thereby further decreasing cellular apoptosis [29]. Collectively, these mechanisms indicate that Klotho confers multiple protective effects in periodontitis, including anti-inflammatory, antioxidant, and anti-apoptotic actions. These effects are achieved by maintaining hPDLSCs’ function and inhibiting oxidative stress and mitochondrial damage, thus presenting a potential molecular target for periodontal tissue regeneration (Table 1).

It is worth noting that the Nrf2/Keap1 signaling pathway acts as a double-edged sword. Its effects are influenced by various factors such as tissue type, disease stage, and underlying molecular alterations [30,31]. On the one hand, the activation of this pathway has been shown to exert protective effects, such as suppressing the progression of periodontitis by modulating inflammation and oxidative stress [30]. On the other hand, accumulating evidence suggests that an aberrant or prolonged activation of the Nrf2/Keap1 pathway is linked to the development and progression of several cancers, including renal cell carcinoma, where it correlates with poor prognosis and resistance to chemotherapy [31]. Due to the complexity of the Nrf2/Keap1 signaling cascade, the precise regulation of Klotho is required to optimize clinical outcomes and minimize adverse reactions. Further investigation into the mechanisms governing the Nrf2/Keap1 pathway will be essential for developing targeted interventions in both inflammatory and oncological diseases.

### 4.2. Role of Klotho in Periapical Disease

Periapical periodontitis is a common oral disease with a reported prevalence of up to 52% at the individual level [32]. The pathology is characterized by the inflammatory destruction of periapical tissues induced by a dynamic imbalance between the microbial community and the host immune system. Although the pathogenicity of microbial agents has been well-established, the precise molecular mechanisms by which dysregulated host immune responses contribute to disease progression remain incompletely elucidated. Generally, various cell types, including periodontal fibroblasts, immune cells, and stem cells, are recognized to play critical roles in defense and reparative processes. Recently, stress-induced senescence has been identified as a factor in numerous inflammatory diseases, leading to impaired tissue function and disease progression. This process is mediated by mechanisms involving oxidative stress, mitochondrial dysfunction, DNA damage, oncogene expression, and the loss of tumor suppressor genes.

It has been demonstrated that cellular senescence is present in clinical periapical lesions, with the Akt/FoxO1 signaling pathway being implicated in H_2_O_2_-induced cellular senescence [33]. Moreover, PI3K has been shown to reduce expression levels of Klotho, phosphorylated Akt, and FoxO1. The FoxO family is known to regulate key physiological functions related to cellular metabolism, growth, differentiation, oxidative stress, autophagy, and senescence. Notably, the activity of FoxO1 is tightly controlled by a phosphorylation-dependent shuttling mechanism mediated via the PI3K-Akt pathway. For example, the Akt-mediated phosphorylation of FoxO1 triggers its nuclear export, thereby modulating the balance between stem cell self-renewal and apoptosis [34].

The *Klotho* gene has been reported to suppress oxidative stress responses by upregulating antioxidant genes such as *CAT* and *SOD*. In studies, early exposure to H_2_O_2_ was found to induce an upregulation of Klotho expression. However, with persistent oxidative damage, Klotho expression was significantly reduced, concomitant with an enhanced senescent phenotype [35]. Further research revealed that the inhibition of Akt/FoxO1 phosphorylation significantly mitigated H_2_O_2_-induced senescence in hPDLSCs [33]. Additionally, the transcriptional regulator PTRF, a key factor in senescence, has been shown to exhibit tissue-specific expression in periapical cysts, suggesting that Klotho may be involved in the oxidative stress-induced senescence process, contributing to periapical disease. Mechanistic studies have indicated that H_2_O_2_ stimulation activates the Klotho–PI3K/Akt/FoxO1 signaling axis, whereas PI3K inhibitors concurrently suppress the expression of both genes and proteins in this pathway. Furthermore, a biphasic model of oxidative stress regulation has been proposed, wherein short-term stress activates FoxO-mediated metabolic adaptation via the inhibition of Akt activity, while sustained damage triggers cellular senescence or apoptosis via the Akt-mediated phosphorylation of FoxO [36]. Collectively, these findings suggest that Klotho may be implicated in the development of periapical periodontitis through the PI3K/Akt/FoxO1 signaling pathway. However, further investigations using animal models are warranted to elucidate its definitive role in systemic homeostasis (Table 2).

### 4.3. Role of Klotho in Oral Submucous Fibrosis

Oral submucous fibrosis (OSF) is an oral mucosal disorder with a predisposition for malignant transformation. The pathological manifestations of OSF are primarily characterized by the degeneration of collagen fibers within the connective tissue of the oral mucosa. In the early stages, fine collagen fibers accompanied by neutrophil infiltration are observed. As the disease progresses, the hyaline degeneration of collagen fibers may occur, along with lymphocytic and plasma cell infiltration, and, in severe cases, extensive myofiber necrosis may be observed. The pathogenesis of OSF is understood to involve an interplay among inflammatory responses, oxidative stress, and fibrotic processes, with various cytokines modulating fibroblast activity and thereby contributing to disease progression [36].

With regard to inflammatory regulation, mediators such as IL-1, IL-6, IL-8, IL-12, TNF-α, interferon (IFN)-γ, inducible nitric oxide synthase (iNOS), and NF-κB have been implicated in the inflammatory response. It has been reported that serum IL-12 levels are inversely correlated with Klotho protein and in vitro experiments have confirmed a bidirectional regulatory relationship between the two [37]. Moreover, IL-12 has been shown to induce iNOS and activate NF-κB, suggesting that Klotho may be involved in the inflammatory process of OSF via the modulation of the IL-12/NF-κB/iNOS signaling axis [38].

In the context of oxidative stress, the Nrf2 pathway and its downstream effectors, HO-1 and NQO1, are recognized as key protective components. An overexpression of Klotho has been demonstrated to significantly activate Nrf2 signaling and promote the expression of SOD2 and NQO1, thereby mitigating oxidative damage in OSF by enhancing the antioxidant defense system [17].

Regarding fibrosis, it has been reported that the inhibition of CpG methylation in the *Klotho* gene promoter induces *Klotho* expression, which subsequently suppresses TGF-β signaling and alleviates renal fibrosis [39]. Consequently, it may be deduced that Klotho is also implicated in the fibrogenic process of OSF (Table 3).

### 4.4. Role of Klotho in Oral and Maxillofacial Malignancies

#### 4.4.1. Role of Klotho in Oral Squamous Cell Carcinoma

In recent years, head and neck cancer (HNC) has been ranked as the sixth-most common malignancy worldwide, with approximately 90% of cases classified as head and neck squamous cell carcinoma (HNSCC) [40]. Despite continuous advancements in treatment modalities, the 5-year postoperative survival rate for HNSCC patients remains below 50% [41]. However, early diagnosis has been associated with a 5-year survival rate of up to 80% [42].

An analysis of The Cancer Genome Atlas (TCGA) database revealed that Klotho expression levels are positively correlated with overall survival in HNSCC patients, whereas promoter methylation levels are inversely correlated [43]. These findings indicate that both Klotho expression and promoter methylation may serve as potential prognostic biomarkers for HNSCC. Moreover, a study demonstrated that the overexpression of Klotho significantly reduced the proliferative activity of Cal-27 and SCC-9 tongue squamous cell carcinoma cells and promoted apoptosis via the inhibition of the Wnt/β-catenin signaling pathway [44].

A series of studies have indicated that epigenetic alterations of the anti-aging gene *Klotho* are emerging as a focus in cancer research. A downregulation of the *Klotho* gene has been observed in various malignancies [45,46], a phenomenon that is primarily attributed to the hypermethylation of CpG islands in its promoter region [45]. DNMTs, which are key enzymes in promoter methylation, have been shown to downregulate tumor suppressor genes through overexpression in cancers, including oral cancer. One study was the first to confirm a significant negative correlation between DNMT3a and *Klotho* expression in oral squamous cell carcinoma cells, suggesting that DNMT3a-mediated hypermethylation and the subsequent epigenetic silencing of the *Klotho* gene may contribute to the pathogenesis of oral and maxillofacial tumors [47]. Notably, both Nicotinamide N-methyltransferase (NNMT) and DNMT are classified within the methyltransferase family and have been demonstrated to play critical roles in cellular metabolism and epigenetic regulation through their enzymatic methylation activities. It has been demonstrated that the upregulation of NNMT is closely associated with oral cancer [48]. However, it is not yet clear whether NNMT influences tumor progression by regulating the anti-aging or metabolic pathways mediated by Klotho protein. Further clarifying the interaction mechanism between Klotho and NNMT in oral cancer may provide a theoretical basis for novel targeted therapeutic strategies, thereby improving the clinical predicament of poor prognosis for oral cancer patients.

#### 4.4.2. Role of Klotho in Salivary Gland Malignant Tumors

Mucoepidermoid carcinoma (MEC) of the salivary glands is one of the most common malignant tumors, affecting both children and adults, with a predilection for the parotid gland. The pathological presentation is typically highly differentiated with low-grade malignancy, a prolonged disease course and usually a painless mass. Regional lymph node metastasis is infrequent, and distant metastasis is rare [49].

Despite extensive research on biomarkers for salivary gland malignancies in recent years, an ideal and universally accepted biomarker has yet to be identified. In a preliminary study, Ma et al. [50] identified carcinoembryonic antigen-related cell adhesion molecule 1 (CEA-CAM1) and Klotho as biomarkers with high specificity and sensitivity for salivary gland malignant tumors, noting that Klotho expression was downregulated in MEC. It has also been demonstrated that activation of the insulin/IGF-1 signaling pathway induces tumor cell growth and proliferation. Studies have reported that, in various tumor cell lines, including those derived from breast cancer, non-small cell lung cancer, gastric cancer, colon cancer, and liver cancer, the silencing or downregulation of Klotho has been shown to facilitate the phosphorylation of insulin and IGF-1 receptors, thereby affecting downstream signaling, activating the IGF-1 pathway, and promoting tumor cell proliferation while inhibiting differentiation [51].

Furthermore, research has demonstrated that an overexpression of Klotho negatively regulates the IGF-1 receptor signaling axis, significantly reducing the expression of the proliferation marker Ki-67 and thereby suppressing tumor cell proliferation and invasiveness [52]. Additionally, Ma et al. [50] reported that the combined assessment of Klotho and Ki-67 expression in MEC could serve as a reliable basis for early clinical diagnosis, metastasis monitoring, and prognostic evaluation. Collectively, these findings indicate that Klotho is a critical biomarker in salivary gland malignant tumors. However, further investigation is required to elucidate the specific mechanistic pathways through which Klotho exerts its effects in MEC (Table 4).

### 4.5. Role of Klotho in Oral and Maxillofacial Bone Defect

Bone defects are common conditions resulting from factors such as trauma, tumors, inflammation, congenital malformations, degenerative diseases, and endocrine disorders [53]. Klotho, as a core component of the FGF receptor complex, has been implicated in the regulation of bone defect repair through the modulation of FGF signaling pathways. It was first demonstrated that the dynamic expression of Klotho is observed in human alveolar bone; its deficiency significantly diminishes the osteogenic capacity of Osx-positive mesenchymal progenitor cells and suppresses osteoclast formation, thereby reducing bone turnover. During alveolar bone defect repair, Klotho has been reported to exert a dual function by modulating both the TNF signaling pathway and the receptor activator of nuclear factor-κB ligand (RANKL) expression, which facilitates bone formation while concurrently inhibiting osteoclast activity. Clinical observations have revealed that serum α-Klotho levels in periodontitis patients are inversely correlated with disease severity, with reduced levels potentially exacerbating bone destruction by disrupting calcium–phosphorus metabolism and vitamin D homeostasis—thus suggesting a protective role for Klotho in inflammatory bone damage [54]. In addition, animal studies have revealed that *Klotho-deficient mice* exhibit a dysplasia of the mandibular ascending ramus accompanied by abnormal calcification, indicating that Klotho may be directly involved in the developmental regulation of local bone metabolism [55].

However, the regulation of osteogenic differentiation by Klotho remains controversial. It has been reported that soluble α-Klotho promotes osteogenic differentiation via the activation of early growth response protein 1 (EGR-1), whereas other studies have indicated that Klotho inhibits osteogenesis through the FGFR1/ERK or Wnt/β-catenin pathways. Furthermore, the osteocyte-specific deletion of Klotho has been observed to result in increased bone mass, suggesting that its effects are dependent on both cell type and microenvironment [56,57,58,59]. In the interplay between inflammation and bone metabolism, bone homeostasis is modulated by Klotho through its influence on the interaction between FGF-23 and hypoxia-inducible factor-1α (HIF-1α). It has been demonstrated that HIF-1α directly activates FGF-23 transcription and *Klotho-deficient mice* have been shown to exhibit more pronounced bone resorption and delayed healing in a periapical periodontitis model, thereby confirming its anti-inflammatory role in bone repair [54,60,61].

Animal models have further elucidated the complex regulatory network of Klotho. For instance, OsxCre;Klothofl/fl *mice* have been shown to exhibit increased bone volume due to the dominant suppression of osteoclast activity [54], while, in *Klotho−/− mice*, B cell-mediated osteoclastogenesis is impaired, indicating that bone remodeling is regulated via a multicellular network [62]. Moreover, the expression of Klotho in mesenchymal stem cells, such as hPDLSCs, provides a molecular basis for its role in guiding stem cell fate during bone regeneration [2,63]. Notably, overexpression experiments have revealed a dual effect of Klotho: on the one hand, the upregulation of RANKL that promotes osteogenic differentiation, and, on the other, the inhibition of osteoclastogenesis through the suppression of RANKL expression. This phenomenon may be attributed to its differential regulation of distinct signaling pathways [54].

In summary, Klotho has been positioned as a molecular hub in bone defect repair by integrating FGF signaling, inflammatory responses, and the regulation of stem cell fate. Its regulatory effects are suggested to differ among various cell types and microenvironments, providing a basis for the contradictory findings observed in some experiments. The targeted modulation of Klotho holds promise as a novel strategy for the repair of alveolar bone defects and the prevention of periodontitis-related bone loss. However, further investigation is warranted to elucidate the underlying mechanisms responsible for the controversial effects of Klotho on osteogenesis, and validation should be pursued using more systematic experimental models, including those involving craniofacial bones (Table 5).

## 5. Discussion

Klotho, an essential anti-aging protein, plays a critical role not only in aging-related diseases but also in the pathogenesis of oral and maxillofacial disorders. Its functions in antioxidation, anti-inflammation, anti-apoptosis, and tissue regeneration render it a vital factor for maintaining oral health, particularly in conditions such as periodontitis, apical periodontitis, oral submucous fibrosis, and oral cancer. Klotho modulates oxidative stress responses, inflammatory reactions, cellular senescence, and fibrosis through multiple signaling pathways, demonstrating its unique therapeutic potential. However, the specific mechanisms by which Klotho exerts its effects remain complex and multifaceted; many aspects still require further investigation.

Current research indicates that Klotho interacts with key pathways, including Nrf2, NF-κB, and Wnt/β-catenin, to regulate oxidative stress levels, inflammation processes, and cellular aging, thereby influencing the progression of oral and maxillofacial diseases. Furthermore, Klotho’s involvement in modulating local immune responses, cellular metabolism, and bone metabolism highlights its therapeutic potential for disease prevention and treatment. As research advances, the multifunctional regulatory properties of Klotho provide new therapeutic strategies, such as utilizing its antioxidant and anti-inflammatory effects to alleviate chronic inflammation in periodontitis or adjusting the balance between osteoblasts and osteoclasts to enhance alveolar bone repair.

However, the precise regulatory mechanisms of Klotho in oral and maxillofacial diseases still require further clarification. Future research should focus on the following areas:

(1) In-depth exploration of Klotho’s interaction with signaling pathways in various oral and maxillofacial diseases.

It is established that Klotho plays a role in disease regulation through multiple signaling pathways; however, its function may vary across different conditions. Notably, the mechanisms by which Klotho operates in inflammatory diseases and tumorigenesis could be significantly influenced by pathological characteristics and the surrounding microenvironment. Therefore, future research should focus on investigating the tissue-specific expression of Klotho across diverse diseases, its interactions within specific signaling pathways, and its dynamic changes throughout disease progression.

(2) Evaluation of the clinical feasibility of Klotho as a diagnostic biomarker and therapeutic target for oral diseases.

The expression levels and promoter methylation status of Klotho may serve as novel biomarkers for the early diagnosis and prognosis of oral and maxillofacial diseases. Further clinical studies should evaluate the sensitivity and specificity of Klotho as a biomarker, as well as its potential in early detection, disease monitoring, and personalized treatment.

(3) Development of Klotho-based therapeutic strategies.

The multifunctional regulatory properties of Klotho make it a promising therapeutic target. Modulating Klotho expression and function through gene editing technologies or small-molecule drugs may provide new treatment options for oral and maxillofacial diseases. For example, interventions targeting the Klotho-Nrf2 pathway could potentially slow the chronic progression of periodontitis, while regulating the interaction between Klotho and the Wnt/β-catenin pathway may offer novel approaches for oral cancer treatment.

(4) Validation of Klotho’s role in animal models and its clinical translational potential.

While studies have explored the effects of Klotho in cellular and animal models, clinical applications continue to encounter significant challenges. Thus, more animal experiments and clinical trials are necessary to validate the therapeutic effects and safety of Klotho in various oral diseases, as well as to assess its feasibility for clinical translation.

## 6. Conclusions

In conclusion, Klotho, as a key biological regulatory factor, holds broad application potential. Future research will help to reveal its full role in oral and maxillofacial diseases, offering new strategies for early diagnosis and precise treatment. With continued advancements in basic research, it is expected that the clinical application of Klotho will garner more attention and bring new hope and treatment options to patients.

## Figures and Tables

**Table 1:** The potential role of Klotho in periodontitis.

Disease	Targets	Results	Models	Ref.
Peridontitis	Nrf2/HO-1	enhance anti- inflammation effects	*mouse* peritoneal macrophages cells, *Sprague−Dawley rats*	[27,28]
	SOD-1	enhance anti- inflammation effects	*mouse* peritoneal macrophages cells, *Sprague−Dawley rats*	[27]
	CAT	enhance anti- inflammation effects	*mouse* peritoneal macrophages cells, *Sprague−Dawley rats*	[27]
	NQO1	enhance anti- inflammation effects	*mouse* peritoneal macrophages cells, *Sprague−Dawley rats*	[27]
	TNF-α, IL-6	alleviate inflammatory response	macrophage cells	[27]
	NF-κB pathway	attenuate pro-inflammatory mediator release	macrophage cells	[27]
	PI3K/Akt/FoxO1	alleviate H_2_O_2_-induced oxidative stress	*human* periodontal ligament stem cells (hPDLSCs)	[15,28]
	Bcl-2	enhance anti- apoptotic effect	*human* periodontal ligament stem cells (hPDLSCs)	[15,28]
	Bax, Caspase-3	enhance anti-apoptotic effect, attenuate H_2_O_2_-Induced apoptosis	*human* periodontal ligament stem cells (hPDLSCs)	[15,28]
	UCP2	preserve mitochondrial function and reduce generation of ROS	*human* periodontal ligament stem cells (hPDLSCs)	[28]

**Table 2:** The potential role of Klotho in periapical disease.

Disease	Targets	Results	Models	Ref.
Periapical Disease	PI3K/Akt/FoxO1	inhibit H_2_O_2_-induced cellular senescence and nuclear translocation	*human* periodontal ligament cells (hPDLCs)	[30,31,33]
	CAT, SOD	inhibit oxidative stress	*human* renal proximal tubular epithelium (HK-2) cells	[32]

**Table 3:** The potential role of Klotho in oral submucous fibrosis (OSF).

Disease	Targets	Results	Models	Ref.
Oral Submucous Fibrosis (OSF)	IL-6, IL-12	alleviate inflammatory response	*C57BL/6-db/db, inbred C57BL/* *6 mice*	[34,35]
	iNOS, NF-κB	alleviate inflammatory response	*mouse* microglia and astrocytes, mouse macrophages	[35]
	Nrf2	attenuated HG- induced oxidative stress and apoptosis	*mouse* podocytes, *C57BLKS/J-LepR (db/db) mice and db/m mice*	[16]
	SOD2, NQO1, HO-1	attenuated HG- induced oxidative stress and apoptosis	*mouse* podocytes, *C57BLKS/J-LepR (db/db) mice and db/m mice*	[16]
	CpG methylation	suppress TGF-β signaling	HK-2 *human* proximal tubule epithelial cell, *C57BL/6 mice*	[36]

**Table 4:** The potential role of Klotho in oral and maxillofacial malignancies.

Disease	Targets	Results	Models	Ref.
Oral Squamous Cell Carcinoma	Wnt/β-catenin pathway	promote the expression of apoptosis- associated proteins	*mouse* peritoneal macrophages cells, *Sprague-* *Dawley rats*	[41]
	DNA methyltransferases (DNMT)3a	downregulate gene expression of hypermethylation of the promoter region	*mouse* peritoneal macrophages cells, *Sprague-* *Dawley rats*	[40,44]
Salivary Gland Malignant Tumors	insulin/IGF-1 pathway	inhibit tumor cell proliferation	MCF-7 breast cancer cells, cervical cancer cells	[47,48]
	Ki-67	inhibit tumor cell proliferation and invasive potential	MCF-7 breast cancer cells, cervical cancer cells	[46,48]

**Table 5:** The potential role of Klotho in oral and maxillofacial bone defect.

Disease	Targets	Results	Models	Ref.
Oral and Maxillofacial Bone Defect	TNF	alleviate inflammatory response	*Klothofl/fl mice* and *Klotho−/− mice*	[50]
	RANKL	promote osteogenesis, alleviate inflammatory response	*Klothofl/fl mice* and *Klotho−/− mice*	[50]
	EGR-1	promote osteogenic differentiation	HEK293 and MC3T3 cells	[52]
	FGFR1/ERK	alleviate inflammatory response	MC3T3-E1 cells, Dmp1-Cre:Klothofl/fl; tdTomatofl/+ *mice* Dmp1-Cre:Klotho−/−; tdTomatofl/ + *mice*	[53]
	Wnt/β-catenin pathway	alleviate inflammatory response	MC3T3-E1 cells, Dmp1-Cre:Klothofl/fl; tdTomatofl/+ *mice* Dmp1-Cre:Klotho−/−; tdTomatofl/ + *mice*	[53]
	FGF	promote osteogenesis	*Klothofl/fl mice* and *Klotho−/− mice*	[50,55,56]

## Data Availability

Not applicable.
